# Decision aid and cost compensation influence uptake of PSA-based early detection without affecting decisional conflict: a cluster randomised trial

**DOI:** 10.1038/s41598-021-02696-z

**Published:** 2021-12-06

**Authors:** Dorothee Tiedje, Matthias Borowski, Alexandra Simbrich, Kathrin Schlößler, Klaus Kruse, Christiane Bothe, Katrin Kuss, Charles Christian Adarkwah, Peter Maisel, Ralf Jendyk, Marc-André Kurosinski, Joachim Gerß, Christian Tschuschke, Ralf Becker, Monique J. Roobol, Chris H. Bangma, Hans-Werner Hense, Norbert Donner-Banzhoff, Axel Semjonow

**Affiliations:** 1 Prostate Center, University Hospital Muenster, Muenster, Germany; 2 Institute of Biostatistics and Clinical Research, University of Muenster, Muenster, Germany; 3 Institute of Epidemiology and Social Medicine, University of Muenster, Muenster, Germany; 4 Department of General Practice/Family Medicine, Philipps-University Marburg, Marburg, Germany; 5 Department of General Practice/Family Medicine, Ruhr-University Bochum, Bochum, Germany; 6 Department of General Medicine, University Hospital Muenster, Muenster, Germany; 7 Berufsverband der Deutschen Urologen, Landesverband Westfalen-Lippe, Muenster, Germany; 8 Hausaerzteverbund Muenster, Muenster, Germany; 9 Department of Urology, Erasmus University Medical Centre, Cancer Institute, Rotterdam, The Netherlands

**Keywords:** Disease prevention, Public health, Prostate, Urological cancer

## Abstract

International guidelines recommend to inform men about the benefits and harms of prostate specific antigen (PSA) based early detection of prostate cancer. This study investigates the influence of a transactional decision aid (DA) or cost compensation (CC) for a PSA test on the decisional behaviour of men. Prospective, cluster-randomised trial to compare two interventions in a 2 × 2 factorial design: DA versus counselling as usual, and CC versus noCC for PSA-testing. 90 cluster-randomised physicians in the administrative district of Muenster, Germany recruited 962 participants aged 55–69 yrs. in 2018. Primary endpoint: the influence of the DA and CC on the decisional conflict. Secondary endpoints: factors which altered the involvement of the men regarding their decision to take a PSA-test. The primary endpoint was analysed by a multivariate regression model. The choice to take the PSA test was increased by CC and reduced by the DA, the latter also reduced PSA uptake in men who were offered CC. The DA led to an increase of the median knowledge about early detection, changed willingness to perform a PSA test without increasing the level of shared decision, giving participants a stronger feeling of having made the decision by themselves. The DA did not alter the decisional conflict, as it was very low in all study groups. DA reduced and CC increased the PSA uptake. The DA seemed to have a greater impact on the participants than CC, as it led to fewer PSA tests even if CC was granted.

**Trial registration**: German Clinical Trial Register (Deutsches Register Klinischer Studien DRKS00007687). Registered: 06/05/2015. https://www.drks.de/drks_web/navigate.do?navigationId=trial.HTML&TRIAL_ID=DRKS00007687.

There is uncertainty as to which men benefit from prostate specific antigen (PSA) based early detection of prostate cancer (PCa). International guidelines recommend that interested men should be informed about the pros and cons before deciding whether or not to take a PSA test^1,2^. A general ambiguity of men and a lack of time available in clinical practice as well as certain preconceptions of physicians with regard to providing this information constitute potential obstacles for establishing balanced decisions^3^. Structured procedures of shared decision making (SDM) may support men in such a situation^4^. While an autonomous and well informed decision remains the goal, new information and individual balancing of options might trigger feelings of uncertainty in men.

This cluster randomised prospective study (PSAInForm) used a computer-based decision aid (DA) for PSA-based early detection of PCa.

As a primary hypothesis, we investigated whether the use of a DA or cost compensation (CC) for a PSA test had an influence on the decisional conflict. According to O'Connor et al., patients counselled with DA are more informed, have more realistic expectations about the possible effects of therapy, report fewer decision conflicts and are more actively involved in the decision-making process^5^. Overall, patients advised in this way tend to adopt a more conservative, less invasive approach. A systematic review of 18 controlled studies shows that decision aids lead to greater knowledge about the disease and reduce the intention to participate in PSA screening^6^. We therefore see the need to conduct research on the acceptance and impact of DA under the specific conditions in Germany.

As in most nations, no population-based prostate cancer screening with PSA is performed in Germany. For opportunistic screening, the statutory health insurances in Germany do not cover the costs for a PSA determination^7^. However, there exists controversy about introduction of such a coverage and we investigated the possible influence this could have on the screening behaviour of men.

As secondary research question, we investigated the influence of the DA on SDM, on the individual’s knowledge, and on the decision for or against PSA uptake. In addition to DA and CC, we investigated other factors that may have an influence on subjects' decision for or against PSA-based early detection, such as the physicians’ attitude or prior PSA experience.

We chose a cluster randomisation in order to avoid that participating physicians had to change their consultation strategies according to the randomisation of patients, which would have introduced an unnecessary contamination.

## Methods

### Study physicians and participants

The study physicians were general practitioners (GPs) or urologists in outpatient setting of the administrative district of Muenster, Germany. They had been invited by the principal investigator (A. Semjonow) to collaborate in the PSAInForm study, taking into account an even distribution (urban/ rural), provided that they self-reported a neutral attitude toward PSA-based early detection. Before joining the study, all study physicians completed a one-hour introduction to familiarise them with the details of the study procedures. At the end of this introduction, physicians were randomly allocated to the clusters after they gave their consent for participation.

Men were invited to enter the study while visiting a study physician’s practice for any kind of health problem, in the frame of disease management programs, for general check up investigations, or if they were interested in undergoing a PSA-test. They had to be aged 55 to 69 according to the core age group of the European Randomised Study of Screening for Prostate Cancer (ERSPC)^8^ and had no history of PCa. Prior PSA testing was no exclusion criterion, but was documented ^9^.

### Trial design

PSAInForm is a prospective, cluster-randomised trial with a 2 × 2 factorial design: GPs and urologists’ practices (clusters) were randomly allocated to one of four study arms (Fig. 2) with differing approaches. To obtain an approximately equal number of practices per arm, the practices were randomised in blocks of four. The DA was used in arms A (with CC) and B (without CC = noCC), whereas in the remaining two arms no DA (noDA) was used: in arm C with and in arm D without CC. Participating men received the type of consultation depending on the randomisation arm of their physician (cluster-randomisation). The participants were asked to fill in questionnaires directly before (T_B_) and directly after (T_0_) the consultation, followed by two telephone interviews two weeks (T_1_) and six months (T_2_) later. During the telephone interviews, the participants were asked if they stuck to their decision regarding the PSA test or not (after 2 weeks) and whether the test was actually performed (within 6 months after consultation).

The 2 × 2 factorial design—use of a computer-based DA or consultation as usual with or without CC—was chosen in order to investigate the possible influences of DA or CC separately.

### Decision aid (DA)

The DA “arriba-PSA” is one module of the library “arriba-lib” that contains several electronic DAs for the diagnosis, prevention and treatment of diseases (eg. cardiovascular prevention or atrial fibrillation) developed on best available evidence^9–11^. These modules are explicitly designed for a use during the consultation between physician and patient (“transactional”). As recommended by the International Patient Decision Aids Standards (IPDAS) the DA was designed and pre-evaluated within a multiple-step mixed-methods pilot study at the Department of General Practice Philipps-University Marburg, Germany^9,12,13^. After a qualitative field-test, the DA was modified according to user’s feedback. Within a randomised-controlled pilot-study, we tested feasibility of study procedures.

**Figure 1:**
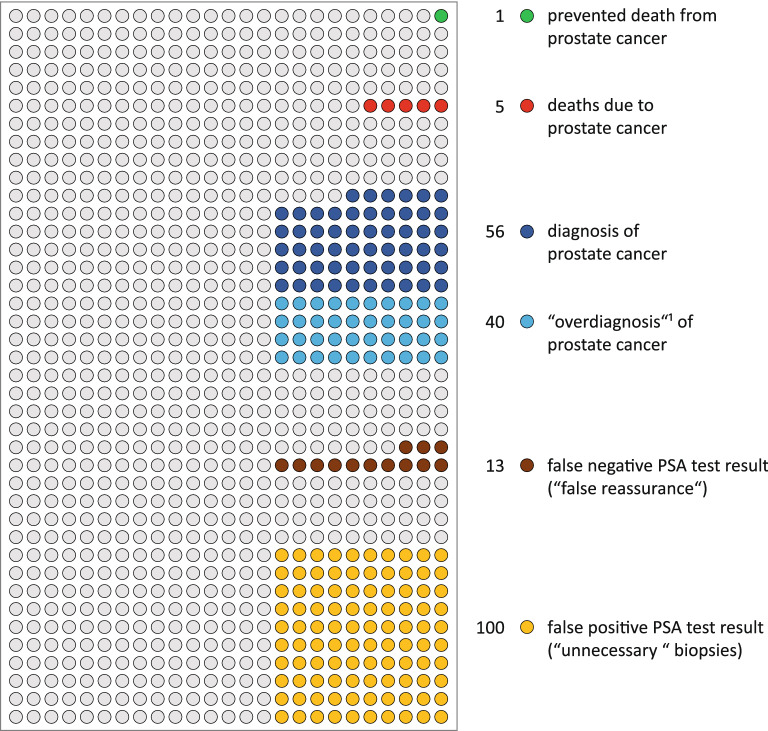
Pictogram of the “arriba-PSA”. It combines information from eight other pictograms included in the transactional DA based on 1000 men (1 circle = 1 man) in the age range of 55–69 years undergoing PSA based early detection of PCa during a period of ten years. It contains information about: dying of PCa within the next ten years; being diagnosed with PCa (risk of a clinically significant vs. not significant cancer) within the next ten years; having false-positive or false-negative PSA test results (risk of “unnecessary” prostate biopsies vs. “false reassurance”) referring to a period of four years only. ^1^Overdiagnosis^16,17^: diagnosis of PCa unlikely to harm the man during his life-time. PSA = prostate specific antigen.

Based on the 10 year results of the core age group of the ERSPC^14^ (55–69 years of age), the DA presents the expected outcome of early detection exemplified for 1000 men. Since the German S3 guideline for early detection of PCa^15^ recommends a PSA concentration of ≥ 4 ng/ml to trigger biopsy, the results of the ERSPC (using a trigger of PSA > 3 ng/ml) were recalculated to a cut off of 4 ng/ml in the initial round of screening by disregarding all men with a biopsy due to a PSA concentration between 3 and < 4 ng/ml. The DA employs tables and pictograms, in which numerical information is presented for 1000 men with versus without PSA-based early detection (Fig. 1)^9^.

### Outcomes

The primary study endpoint was decisional conflict of the participating men as measured by the decisional conflict scale (DCS)^18^ two weeks after consultation (T_1_). The primary study questions were:

1. Does a consultation with the DA influence the decisional conflict regarding PSA?
2. Does CC of the PSA test influence the decisional conflict?

The DCS measures the uncertainty with a decision made^18^ and ranges from “0” (no decisional conflict) to “100” (extremely high decisional conflict)^19^. DCS lower than 25 are associated with the implementation of the decision (e.g. perform or not perform a PSA-test)^18^.

Secondary study endpoints were used to compare the consultation with and without the DA in terms of patient involvement (SDM-Q-9 and own appraisal of involvement in decision making) and knowledge. In addition we analysed which further factors influenced the men’s decision for or against PSA-based early detection of PCa (use of the DA, CC, physicians’ attitude, and prior PSA experience). We measured participants’ decision making by asking them about their own appraisal of involvement. Moreover, they were asked to fill in the questionnaire SDM-Q-9. The SDM-Q-9 consists of nine statements, which can be rated on a six-point likert-type-scale from ‘‘completely disagree’’ (0) to ‘‘completely agree’’ (5). A transformed mean of sum-score reveals values between 0 and 100, where 0 indicates the lowest and 100 the highest level of SDM^20^. We assessed knowledge with a mean of sum score from 1 to 11 according to previous work of Watson et al. Higher values indicate better knowledge^21^. Influence of the physician’s attitude towards PSA was estimated by asking whether male physicians already had their own PSA determined or if female physicians advised relatives to have a PSA determination. It was documented which men opted for taking the PSA test at T_0_ and whether they had actually performed the PSA test in the meantime at T_1_ and T_2_.

### Statistical analyses

We established an adaptive group-sequential design according to O’Brien-Fleming with one interim analysis to test the primary study questions in a confirmatory manner (information rate 0.5), and to recalculate the sample size using the inverse normal method. The interim analysis was necessary, as the effect of cluster-variations cannot be assessed in advance, and was planned to be performed after recruitment of n = 927 participants.

The primary endpoint in the interim analysis was analysed by a multivariate regression model. Initial sample size calculation was performed assuming an intra-cluster correlation coefficient ICC = 0.1, and a mean cluster size of 12.9 participants per practice based on pilot data. Sample size calculation revealed a power of > 80% to confirm each primary study question hypothesis by a two-sample *t*-test (Welch) if the total sample size in the final analysis was n = 1614 participants in 125 practices and if the effect size in terms of Cohen’s d is d ≥ 0.228. With an expected standard deviation σ≈10, this effect size corresponds to a difference of 2.28 units in the DCS score. Because of the cluster-randomisation, the estimation of regression coefficients and related standard errors were carried out using Generalised Estimating Equations (GEEs), in order to account for intra-cluster correlations of patients who belong to the same physician.

The secondary endpoints were analysed comparing groups of DA/noDA and CC/noCC by Mann–Whitney-U-Test and exact Fisher test. The interaction of CC and the DA was investigated by using a logistic regression model including three binary covariates, eg. “DA” (yes/no), “CC” (yes/no) and the interaction covariate “DA and CC” and the exact Fisher test. The association of physicians’ attitude and participants’ decision to undergo a PSA test was measured by exact Fisher test. Bonferroni correction was used to control a global 5% level of significance and allow for testing of the primary and secondary endpoints.

Results are presented graphically by means of Boxplots. The box corresponds to the upper and lower quartile. Whiskers are drawn from the ends of the box to the largest and smallest values that are within 1.5 times the interquartile range from the end of the box.

All statistical analyses whose results are reported here were specified beforehand in a Statistical Analysis Plan for the final analysis*.*

### Ethics approval and consent of participants

The study was approved by “Ethik-Komission der Ärztekammer Westfalen-Lippe und der Westfälischen Wilhelms-Universität”, reference number 2013-367-f-S (vote from 18 September 2015). Participants and physicians gave their informed consent to participate in the study. Research was performed in accordance with the Declaration of Helsinki.

## Results

### Recruitment

**Figure 2:**
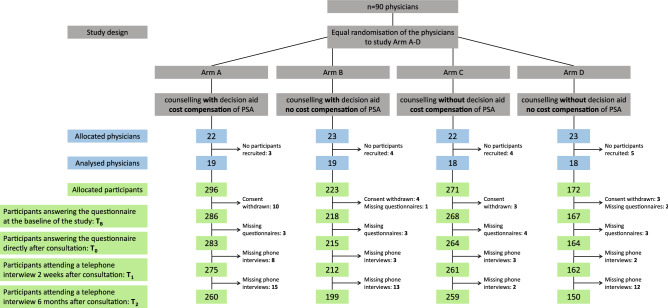
CONSORT flow diagram of the cluster-randomised controlled trial “PSAInForm” with a 2 × 2 factorial design. 90 physicians are randomised to one of four arms (A-D) before they start to recruit participants. PSA = prostate specific antigen.

A total of 90 practices were randomised (68 GPs, 22 urologists) between April 2015 and August 2016 and allocated to the four study arms (Fig. 2) according to our research questions. While 16 practices did not recruit any patients, the remaining 74 practices recruited 962 men between July 2015 and May 2017 (Fig. 2). The highest recruitment numbers were achieved in the arms with CC (arm A and C), while the lowest number of participants was recruited in arm D (noDa-noCC).

### Interim analysis

The interim analysis was performed as scheduled after recruiting 927 participants. Regarding the primary endpoints, there was no clinically relevant impact of the DA or CC on the decisional conflict of the participants at T_1_. To reach a possibly statistically significant, but clinically negligible difference of less than one point on the DCS scale, the sample size recalculation resulted in 3.272 participants. The steering committee stopped the study for futility reasons at this point in time, by which 35 further participants had been recruited, leading to a final total number of 962 participants. Our interim analysis (based on 927 participants) provides type I error control on a 5% significance level and therefore confirmatory statistical evidence. It is a common approach in trials with interim analyses to perform an additional statistical analysis with the total of all recruited participants, in our study n = 962.

### Final analysis

As a consequence, the final analysis tested the primary endpoint only in an explorative manner without type I error control together with the secondary endpoints. Because of only few major protocol deviations there was no difference between “per protocol” and “intention to treat” evaluations; therefore, the results of the intention to treat analysis are presented in the following only.

### Baseline characteristics

The parameters in Table 1 show a high similarity of the participants in all study arms, thus confirming an effective randomisation procedure. It is noteworthy that most of the participants in all study arms had previous experience with early detection, both with PSA uptake and digital rectal examination. The compliance of the participants with the study protocol including follow-up was very high overall and homogeneously distributed in the arms.

**Table 1:** Baseline characteristics of the participants, prior experience of early detection for PCa and compliance with the study protocol.

	Arm A	Arm B	Arm C	Arm D
	DA-CC	DA-noCC	noDA-CC	noDA-noCC
	n = 296	n = 223	n = 271	n = 172
**Baseline characteristics of the participants**				
Median age *years (range)*	61 (55–69)	61 (55–69)	60 (55–69)	61 (55–69)
In relationship *n(%)*	256 (90)	202 (93)	249 (93)	148 (89)
German nationality *n(%)*	279 (98)	216 (99)	266 (99)	167 (100)
Self-assessment of health as “good”^a^* n(%)*	200 (70)	141 (66)	173 (67)	112 (68)
**Prior experience of early detection of prostate cancer**				
DRE only *n(%)*	54 (20)	31 (15)	26 (10)	35 (22)
PSA test only *n(%)*	17 (6)	18 (9)	11 (4)	10 (6)
DRE and PSA test *n(%)*	173 (64)	127 (61)	199 (79)	99 (62)
PSA experience in general *n(%)*	190 (70)	145 (70)	210 (83)	109 (69)
Never had a PSA test *n(%)*	80 (30)	63 (30)	43 (17)	50 (31)
No prior experience with early detection *n(%)*	26 (10)	32 (15)	17 (7)	15 (9)
**Frequency of previous early detection**				
1 *n(%)*	27 (11)	27 (16)	31 (13)	15 (11)
2–3 *n(%)*	76 (31)	65 (37)	61 (26)	31 (22)
> 3 *n(%)*	140 (58)	82 (47)	144 (61)	97 (68)
**Compliance with the study protocol**				
Completed questionnaire at T_0_ *n(%)*	283 (99)	215 (99)	263 (98)	163 (98)
Completed telephone interview at T_1_ *n(%)*	275 (96)	211 (97)	261 (97)	159 (95)
No relevant protocol deviations *n(%)*	272 (95)	207 (95)	258 (96)	152 (91)

^a^5-point scale with answer options: “excellent”, “very good”, “good”, “less good”, “bad”. “Good” was placed in the middle.

*PSA* prostate specific antigen, *DRE* digital rectal examination, *T*_*0*_ directly after consultation, *T*_*1*_ two weeks after consultation.

### Primary endpoint

**Figure 3:**
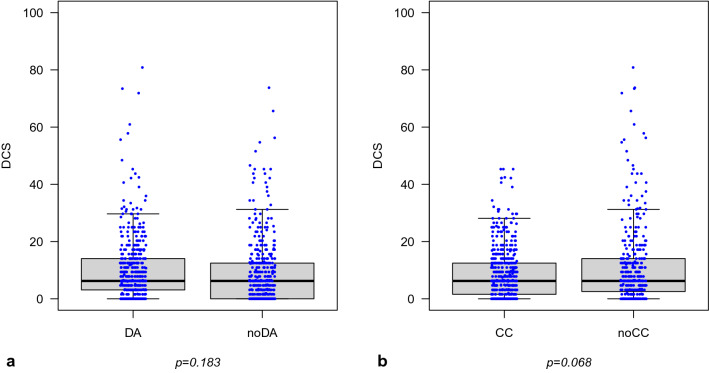
Boxplots comparing the DCS score between study arms. (**a**) Consultation with DA (arm A + B): Q25% = 3.1; Median = 6.2; Q75% = 14.1 vs. consultation without DA = noDA: Q25% = 0; Median = 6.2; Q75% = 12.5. (**b**) Consultation with cost compensation CC (arm A + C): Q25% = 1.6; Median = 6.2; Q75% = 12.5 vs. without cost compensation = noCC (arm B + D): Q25% = 2.5; Median = 6.2; Q75% = 14.1. DCS = decisional conflict scale: 0–100; higher values indicate greater decisional conflict.

Figure 3 depicts the distribution of the DCS scores at T_1_ in the two study groups. The distribution was highly skewed towards low decisional conflict and did not differ significantly between the groups. The median DCS score in each group was 6.2 with similar interquartile ranges (Fig. 3). The intra-cluster correlation coefficient ICC is 0.02.

### Secondary endpoints

#### Comparison of DA and noDA in terms of decision making

Most participants, whether receiving DA or noDA, were actively involved in the decision-making process. Consultation with the DA led to a lower proportion of physicians’ involvement in decisions and was associated with a lower SDM-score than consultation without the DA (Table 2). The proportion of participants who made the decision only by themselves was higher in the DA group than in the noDA group (39% vs. 27%, *p* < 0.001; Table 2). Men exposed to the DA had higher knowledge according to the Watson-score compared to participants in the noDA group (median 8 vs. 7, *p* < 0.001; Table 2).

**Table 2:** Comparison of decision aid (DA) and no decision aid (noDA) regarding shared decision making, knowledge about PSA, decision concerning PSA test and distribution according to cost compensation (CC).

	DA (n = 519)	noDA (n = 443)	*p*-value
**Assessment of participants’ decision making** ***n*(%)**	n = 489	n = 415	< 0.001
Only by myself	193 (39)	112 (27)	
Mostly by myself	103 (21)	73 (18)	
By physician and myself	185 (38)	219 (53)	
Mostly by physician	6 (1)	9 (2)	
Only by physician	2 (0)	2 (0)	
**SDM-Q-9-score (0–100) for shared decision making at T0**	n = 490	n = 410	< 0.001
Median	84.4	88.9	
Q25; Q75	64.4; 95.6	71.1; 100	
Range	8.9–100	0–100	
**Participants’ knowledge (Watson-score: 1–11)**	n = 495	n = 420	< 0.001
Median	8	7	
Q25; Q75	6; 9	5; 8	
Range	0–11	0–11	
***Participants’ decision at T***_***1***_ ***n*(%)**	n = 487	n = 423	< 0.001
PSA test	350 (72)	373 (88)	
No PSA test	106 (22)	32 (8)	
No decision	31 (6)	18 (4)	
**PSA test performed until T**_**2**_ ***n*(%)**	n = 457	n = 409	< 0.001
Yes	341 (75)	361 (88)	
No	114 (25)	47 (11)	
Don ‘t know	2 (0)	1 (0)	
***Proportion of men who decided for PSA test according to CC at T***_***1***_ ***n*(%)**			
CC	223 (81)	246 (94)	< 0.001
noCC	127 (60)	127 (78)	< 0.001

*T*_*0*_ directly after consultation, *T*_*1*_ two week after consultation, *T*_*2*_ six months after consultation, *PSA* prostate specific antigen, *noCC* without cost compensation.

#### Influence of DA and noDA on PSA-decision

Although the majority of participants in both groups (DA vs. noDA) decided to take a PSA test, their proportion was significantly lower among men in the DA group (72% vs. 88%, respectively). Consequently, fewer men in the DA group actually performed a PSA test within 6 months (Table 2). Among the participants who received DA *and* CC, 81% decided to take a PSA test, indicating that the readiness to undergo the PSA test in the group with DA is increased by CC (Table 2).

Since the majority of participants (n = 654, 73%) had already taken PSA tests before (Table 1), we were interested in potentially differing patterns in the subgroup of men without prior PSA test. In this subgroup (n = 225) the decision to take a PSA test was less frequent altogether and lower with the DA (n = 89, 64%) as compared to noDA (n = 65, 75%), without reaching statistical significance (*p* = 0.08).

Generally, participants in the group with CC were significantly more likely to decide for the PSA test (88% vs. 68%, *p* < 0.001) and to have a PSA test (87% vs. 73%, *p* < 0.001; Table 3) as compared to noCC.

**Table 3:** Comparison of cost compensation (CC) and no cost compensation (noCC) regarding the decision and performance of a PSA test.

	CC (n = 567)	noCC (n = 395)	*p*-value
**Participants’ decision at T**_**1**_ ***n*(%)**	n = 536	n = 374	< 0.001
PSA test	469 (88)	254 (68)	
No PSA test	53 (10)	85 (23)	
No decision	14 (3)	35 (9)	
**PSA test performed until T**_**2**_ ***n*(%)**	n = 519	n = 347	< 0.001
Yes	450 (87)	252 (73)	
No	66 (13)	95 (27)	
Don’t know	3 (1)	0 (0)	

*T1* two week after consultation, *T*_*2*_ six months after consultation, *PSA* prostate specific antigen.

#### Influence of physician’s attitude on PSA-decision

Furthermore, the correlation of the physicians’ attitude regarding PSA tests on participants’ decision to undergo a PSA test was analysed for all participants. Most urologists (20/22) and GPs (49/67) had a positive attitude towards PSA testing. Participants advised by physicians with positive attitude (n = 774, 82%) decided more often to undergo a PSA test (n = 619, 82%) than participants counselled by physicians without positive attitude (n = 104, 66%); *p* < 0.001.

## Discussion

Investigating the influence of a DA and CC on the decisional behavior of men interested in PSA based early detection, the choice to take the PSA test was increased by cost compensation for the test and lowered by offering information about the pros and cons of early detection in this randomised trial.

### Primary endpoint: decisional conflict

The low DCS-score in all four study groups (median: 6.2) suggests that a decisional conflict concerning PSA based PCa screening does hardly exist in our study population. This may explain why the DA in our study did not result in changes of decisional conflicts, thus eventually rendering the study as futile. Nevertheless, some reports seem to show that a DA can reduce the decisional conflict concerning PCa screening, despite low DCS-scores^4,22,23^. Our finding, that cost compensation neither increases nor decreases the decisional conflict to perform a PSA test could be of interest as an argument for a possible future cost coverage of the PSA test by health insurances in Germany.

### Secondary endpoints

#### Influence of DA on shared decision making

Participants with DA attributed their decision more to themselves than to the influence of the physician although it was a participative DA. They seem to be empowered to meet their own decision autonomously. Also the review of Volk et al. reported, that DAs promote participants to become more likely to take an active role in decision making and less likely to defer control to their physicians^6^. This is in line with lower SDM-scores in the DA group. We interpret lower SDM-scores according to a higher patient autonomy. However, SDM-9-Scores were high in the noDA-groups as well, which might indicate a shift away from the paternalistic consultation model by the arriba-DA^24^. We could confirm the finding of Sheridan et al. that a SDM intervention using a DA does not necessarily result in increasing shared decisions^25–27^.

Despite lower SDM scores in the DA group and a generally high willingness to take a PSA test in our 4 study groups, the proportion to undergo a PSA test was significantly lower in men counselled with DA, confirming results from Sheridan et al. that DA decreased the willingness to undergo a PSA test without increasing the level of shared decisions. Our study showed that the DA led to fewer PSA tests, which has also been described in several reviews^6,23,26,28^. Nevertheless, a recent meta-analysis by Riikonen et al. did suggest that screening decisions are possibly not associated with DAs^22^.

#### Influence of the DA on knowledge

The use of the DA in this study increased the median knowledge score confirming previous reports^6,22,23,28,29^. However, no group did reach excellent knowledge scores directly after the consultation. We used a score that was not directly linked to the DA according to wording and sequence, which might contribute to the rather modest increase of knowledge. Nevertheless, we interpret the difference between DA and noDA as relevant.

#### Influence of the DA with or without CC on men’s decision for or against the PSA-test

We noted that CC for the PSA test increased the decision for the test irrespective of the application of DA, which seems to suggest that CC generally raises the motivation to take the test. On the other side, DA reduced willingness to take the test by a similar proportion also when CC was offered, showing that this effect was generally independent from cost aspects.

Results in the subgroup of PSA-naive men generally confirmed these findings. In these participants the proportion taking PSA tests was as low as 64% in the DA group.

It appears that the use of a DA had a stronger influence on the decision to perform a PSA test than CC.

#### Influence of the physician

Although we required that physicians had a self-reported neutral attitude towards PSA screening when joining the study, the test was more likely to be performed if the consultation took place with a physician who had a more positive attitude towards PSA, which has been described already^30^.

## Strengths and limitations

The strength of this prospective randomised study is the large sample size with high compliance and no major protocol deviations. It is limited by the fact that most of the participants had previous experience of early detection (90%), with PSA (73%) or DRE only (17%).

The recruitment in arm D (noDA-noCC) resulted in a lower number of participants. Possible reasons could be that the motivation of the physicians to recruit participants and the willingness of the participants to join the study was reduced because nothing more than the usual was offered.

A methodological limitation may have occurred as several participants perceived many questions of the DCS questionnaire as synonymous. Beyond that, understanding the implications of cancer screening, complex numerical information and abstract concepts must be illustrated. As we learnt from informal feedback from study participants, this provides a challenge for patients and clinicians alike.

For the transferability of our study results to other countries, the existing regulation of cost coverage for early detection measures must be taken into account.

## Conclusions

The large majority of study participants in this cluster-randomised trial chose to undergo a PSA based early detection of PCa. The choice to take the PSA test was higher in groups with CC while it was lower in groups using the DA. The DA also reduced the uptake of PSA tests, if CC was offered. Application of the DA led to greater knowledge and a reduced willingness to perform a PSA test, but without raising the level of shared decisions. Rather, the DA tended to give participants the feeling that they had done a self-empowered decision. Generally, the level of decisional conflict was very low in all study groups and DA appeared not to affect it. In our view, the finding that counselling by DA did not alter the decisional conflict should not prevent men from being informed about the advantages and disadvantages of prostate cancer early detection before deciding for or against. The use and further refinement of DAs should remain a high priority. Ideally, screening should be discussed before it is performed for the first time. Implication of our study together with available evidence in literature suggests that decision aids compared with usual care may or may not be associated with a minor decrease in decisional conflict, but may increase knowledge regarding early detection of PCa. With growing evidence concerning the pros and cons of PCa early detection, decision aids should incorporate the continuously updated results of randomised screening trials in terms of mortality reduction and the risk of overdiagnosis. The finding that cost compensation showed no influence on the decisional conflict and that the use of the decision aid leads to fewer PSA uptake regardless of cost compensation should be taken into account when considering future reimbursement by statutory health insurance.

## Data Availability

Data collected for the study, including deidentified individual participant data and a data dictionary defining each field in the set, will be made available to others. Related documents will be available (e.g. study protocol, statistical analysis plan, informed consent form). These data will be available with publication, proposals may be submitted up to 5 years following publication. Data will be shared with interested bodies including investigator support after approval of a proposal with a signed data access agreement. Proposals should be directed to the corresponding author.

## Abbreviations

CC: Cost compensation
DA: Decision aid
DCS: Decisional conflict scale
ERSPC: European Randomised Study of Screening for Prostate Cancer
GEEs: Generalised estimating equations
GPs: General practitioners
IPDAS: International Patient Decision Aids Standards
ICC: Intra-cluster correlation coefficient
noCC: No cost compensation
noDA: No decision aid
PCa: Prostate cancer
PSA: Prostate specific antigen
SDM: Shared decision making

## References

[1] Carter HB (2013). Early detection of prostate cancer: AUA guideline. J. Urol..

[2] Mottet N (2017). EAU-ESTRO-SIOG guidelines on prostate cancer. Part 1: Screening, diagnosis, and local treatment with curative intent. Eur. Urol..

[3] Tiedje D (2017). Anwendung der S3-Leitlinie zur Prostatakrebsfrüherkennung in urologischen Praxen. [Use of the S3 guidelines for early detection of prostate cancer in urological practices]. Urologe A..

[4] Krist AH, Woolf SH, Johnson RE, Kerns JW (2007). Patient education on prostate cancer screening and involvement in decision making. Ann. Fam. Med..

[5] O'Connor AM (2009). Decision aids for people facing health treatment or screening decisions. Cochrane Database Syst. Rev..

[6] Volk RJ (2007). Trials of decision aids for prostate cancer screening: A systematic review. Am. J. Prev. Med..

[7] Simbrich A, Semjonow A, Donner-Banzhoff N, Hense HW (2018). Praxis der Früherkennung des Prostatakarzinoms: Deskriptive Erhebung im Vorfeld der PSAInForm-Studie. [Practice of early detection of prostate cancer : Descriptive survey in preparation for the PSAInForm study]. Urologe. A..

[8] Schroder FH (2009). Screening and prostate-cancer mortality in a randomized European study. N. Engl. J. Med..

[9] Semjonow A (2019). Development and prospective randomized evaluation of a decision aid for prostate-specific antigen-based early detection of prostate cancer in men aged between 55 and 69yr: The PSAInForm Trial. Eur. Urol..

[10] Hirsch O, Keller H, Krones T, Donner-Banzhoff N (2011). Acceptance of shared decision making with reference to an electronic library of decision aids (arriba-lib) and its association to decision making in patients: an evaluation study. Implement. Sci..

[11] Krones T (2008). Absolute cardiovascular disease risk and shared decision making in primary care: A randomized controlled trial. Ann. Fam. Med..

[12] Volk RJ, Llewellyn-Thomas H, Stacey D, Elwyn G (2013). Ten years of the International Patient Decision Aid Standards Collaboration: Evolution of the core dimensions for assessing the quality of patient decision aids. BMC Med. Inform. Decis. Mak..

[13] Kuss K (2021). Delivering the unexpected-Information needs for PSA screening from Men's perspective: A qualitative study. Health Expect..

[14] Schroder FH (2014). Screening and prostate cancer mortality: Results of the European randomised study of screening for prostate cancer (ERSPC) at 13 years of follow-up. Lancet.

[15] Leitlinienprogramm Onkologie (Deutsche Krebsgesellschaft, Deutsche Krebshilfe, AWMF): Interdisziplinäre Leitlinie der Qualität S3 zur Früherkennung, Diagnose und Therapie der verschiedenen Stadien des Prostatakarzinoms, Leitlinienreport, Version 5.1, 2019. AWMF-Registernummer: 043/022OL. (accessed 19 Mar 2021); https://www.leitlinienprogramm-onkologie.de/leitlinien/prostatakarzinom (2019).

[16] Heijnsdijk EAM (2018). Summary statement on screening for prostate cancer in Europe. Int. J. Cancer.

[17] Draisma G (2009). Lead time and overdiagnosis in prostate-specific antigen screening: importance of methods and context. J. Natl. Cancer Inst..

[18] O’Connor A. User Manual: Decisonal Conflict Scale (accessed 19 Mar 2021); http://decisionaid.ohri.ca/docs/develop/User_Manuals/UM_Decisional_Conflict.pdf. (2005).

[19] Linder SK (2011). Validity of a low literacy version of the decisional conflict scale. Patient Educ. Couns..

[20] Kriston L (2010). The 9-item Shared decision making questionnaire (SDM-Q-9). Development and psychometric properties in a primary care sample. Patient Educ. Couns..

[21] Watson E (2006). Informed decision making and prostate specific antigen (PSA) testing for prostate cancer: A randomised controlled trial exploring the impact of a brief patient decision aid on men's knowledge, attitudes and intention to be tested. Patient Educ. Couns..

[22] Riikonen JM (2019). Decision aids for prostate cancer screening choice: A systematic review and meta-analysis. JAMA Int. Med..

[23] Ivlev I, Jerabkova S, Mishra M, Cook LA, Eden KB (2018). Prostate cancer screening patient decision aids: A systematic review and meta-analysis. Am. J. Prev. Med..

[24] Scholl I (2011). Measurement of shared decision making: A review of instruments. Z Evid Fortbild Qual Gesundhwes..

[25] Sheridan SL (2012). Shared decision making for prostate cancer screening: The results of a combined analysis of two practice-based randomized controlled trials. BMC Med. Inform. Decis. Mak..

[26] Stacey D (2017). Decision aids for people facing health treatment or screening decisions. Cochrane Database Syst. Rev..

[27] Légaré F (2018). Interventions for increasing the use of shared decision making by healthcare professionals. Cochrane Database Syst. Rev..

[28] Evans R (2005). Reduction in uptake of PSA tests following decision aids: Systematic review of current aids and their evaluations. Patient Educ. Couns..

[29] Ilic D (2015). Assessing the effectiveness of decision aids for decision making in prostate cancer testing: A systematic review. Psychooncology.

[30] Pucheril, D. *et al.* The influence of physician recommendation on prostate-specific antigen screening. *Urol. Oncol.* **33(10)**, 424 e421–427 (2015). 10.1016/j.urolonc.2015.06.013 26206103

